# A methodology for deriving the sensitivity of pooled testing, based on viral load progression and pooling dilution

**DOI:** 10.1186/s12967-019-1992-2

**Published:** 2019-08-06

**Authors:** Ngoc T. Nguyen, Hrayer Aprahamian, Ebru K. Bish, Douglas R. Bish

**Affiliations:** 1 Grado Department of Industrial and Systems Engineering, Virginia Tech, Blacksburg, VA 24061 USA; 2 Department of Industrial and Systems Engineering, Texas A&M University, College Station, TX 77843 USA

**Keywords:** Sensitivity estimation, Pooled testing, Pooling dilution, Public health screening, Surveillance study

## Abstract

**Background:**

Pooled testing, in which biological specimens from multiple subjects are combined into a testing pool and tested via a single test, is a common testing method for both surveillance and screening activities. The sensitivity of pooled testing for various pool sizes is an essential input for surveillance and screening optimization, including testing pool design. However, clinical data on test sensitivity values for different pool sizes are limited, and do not provide a functional relationship between test sensitivity and pool size. We develop a novel methodology to accurately compute the sensitivity of pooled testing, while accounting for viral load progression and pooling dilution. We demonstrate our methodology on the nucleic acid amplification testing (NAT) technology for the human immunodeficiency virus (HIV).

**Methods:**

Our methodology integrates mathematical models of viral load progression and pooling dilution to derive test sensitivity values for various pool sizes. This methodology derives the conditional test sensitivity, conditioned on the number of infected specimens in a pool, and uses the law of total probability, along with higher dimensional integrals, to derive pooled test sensitivity values. We also develop a highly accurate and easy-to-compute approximation function for pooled test sensitivity of the HIV ULTRIO Plus NAT Assay. We calibrate model parameters using published efficacy data for the HIV ULTRIO Plus NAT Assay, and clinical data on viral RNA load progression in HIV-infected patients, and use this methodology to derive and validate the sensitivity of the HIV ULTRIO Plus Assay for various pool sizes.

**Results:**

We demonstrate the value of this methodology through optimal testing pool design for HIV prevalence estimation in Sub-Saharan Africa. This case study indicates that the optimal testing pool design is highly efficient, and outperforms a benchmark pool design.

**Conclusions:**

The proposed methodology accounts for both viral load progression and pooling dilution, and is computationally tractable. We calibrate this model for the HIV ULTRIO Plus NAT Assay, show that it provides highly accurate sensitivity estimates for various pool sizes, and, thus, yields efficient testing pool design for HIV prevalence estimation. Our model is generic, and can be calibrated for other infections.

## Background

*Pooled testing*, in which biological specimens (e.g., blood, urine, tissue swabs) from multiple subjects are combined into a testing pool and tested via a single test, can substantially improve the efficiency of public health screening and population-level surveillance of diseases; and enables the use of expensive testing technologies, such as the nucleic acid amplification testing (NAT) technology [2]. Ever since its introduction in the 1940’s [9], pooled testing has been commonly used for both surveillance and screening purposes, including donated blood screening for transfusion-transmittable infections, e.g., the human immunodeficiency virus (HIV), or regional HIV surveillance [1, 7].

In general, biomarker tests have less than perfect *sensitivity* (true positive probability), mainly because the progression of the load (concentration) of a disease-related biomarker (e.g., the HIV viral RNA, measured by the NAT) in the infected host follows various phases post-exposure, with varying growth rates, e.g., pre ramp-up phase, ramp-up phase with accelerating growth rates, and post ramp-up phase, with the biomarker load tending to a plateau or significantly diminishing due to a resolved infection (e.g., [10, 20]). A majority of false negative testing errors occur during those earlier phases of the infection (pre ramp-up and early ramp-up phases), also known as the *window period*, the length of which depends on the specific infection and the biomarker being measured by the test (e.g., [27]). Pooled testing may further reduce the test’s sensitivity due to *pooling dilution*, where the biomarker load of an infected specimen is diluted by infection-free specimens in the pool so that the infected specimen may no longer be detectable by the pooled test. Pooled testing sensitivity at various pool sizes is an essential input to key decisions in surveillance and screening efforts, including testing pool design. However, clinical data on test sensitivity values for different pool sizes are limited, and the extant literature that analytically derives the sensitivity of a pooled test does so under restrictive assumptions, including that the test is perfectly reliable outside of the window period, i.e., all infected specimens that are outside of the window period are detected with probability 1 regardless of the pool size (e.g., [4, 27, 28]). There are commonly adopted mathematical models of viral load progression in infected subjects, but these models consider only the window period (e.g., [6]).

Therefore, our objective in this paper is to develop a generic methodology for analytically deriving the sensitivity of pooled testing at various pool sizes, based on models that account for viral load progression and pooling dilution; and by relaxing various restrictive assumptions adopted in the literature. In particular, our methodology integrates the following components within a probabilistic framework: (1) the “doubling time” model [6], which we expand to model the host’s viral load progression throughout the infection’s life-time; and (2) the probit function [27], which we expand to model pooling dilution to consider the number of infected specimens in a pool. The proposed methodology derives the *conditional test sensitivity*, conditioned on the number of infected specimens in a pool; and uses the law of total probability to derive overall (unconditional) test sensitivity values for a wide range of pool sizes. We validate this methodology via published test sensitivity data and show that it is highly accurate. This methodology utilizes higher dimensional integrals, which may be computationally expensive for large pools. As a result, we also propose an easy-to-compute, and a highly accurate, approximation function that is based on establishing a functional relationship between the sensitivity of pooled testing and the number of infected specimens in a pool. Our methodology can be used to provide important inputs for surveillance and screening activities, including testing pool design, which has received considerable attention in the literature, (e.g., [16–18, 25, 26, 30, 31]). Further, our methodology is generic, and can be calibrated for various infections; and we demonstrate its application for the HIV and HIV ULTRIO Plus NAT Assay. For this purpose, we calibrate model parameters using published test efficacy data for the HIV ULTRIO Plus Assay [20, 22], and clinical data on viral RNA load progression in HIV-infected patients [6, 27]; and use this methodology to derive and validate the sensitivity of the HIV ULTRIO Plus Assay for various pool sizes. We also demonstrate the value of this methodology through optimal testing pool design for HIV prevalence estimation in Sub-Saharan Africa. This case study indicates that the optimal testing pool design is highly efficient, and outperforms a benchmark pool design.

## Methods

Our methodology is based on the integration of viral load progression and pooling dilution models. A summary of all the notation used in our study is provided in the Appendix.

### Pooled sensitivity estimation methodology

#### Viral load progression model

**Fig. 1:**
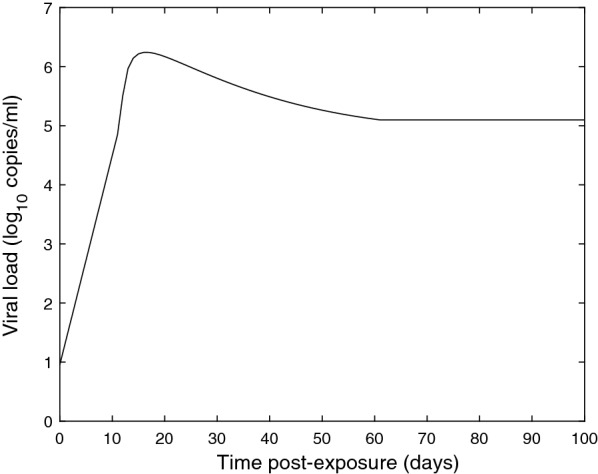
HIV viral RNA load progression spanning the infection’s life-time, covering the window period, peak viremia phase, and chronic phase (based on the data in Table 1)

We first describe the viral load progression model, which expands the widely adopted doubling time model proposed by Busch et al. [6]. The original doubling time model [6] considers viral load progression only through the window period of an infection, and we expand it to model the infection’s life-time. This is needed to relax a common assumption used in test sensitivity calculations, that all infected specimens outside of their window period are detected with probability 1, regardless of the pool size (e.g., [27, 28]). According to numerous studies, the viral load in infected subjects progresses through various phases of growth rates post-exposure: pre ramp-up phase, ramp-up phase with accelerating growth rates, and post ramp-up phase during which growth rate slows down, eventually reaching a plateau or resolution of the infection (e.g., [5, 6, 10, 12, 20, 27, 28]). To model this phenomenon, we let $t_w$, $t_p$, and $t_s$ respectively denote the time at which the window period ends, viral load peaks, and viral load reaches steady state; and let *VL*(*t*) denote the infected subject’s viral load at time *t* post-exposure. Based on clinical data for HIV and hepatitis B and C infections [5, 6, 10, 12], we model the infected subject’s viral load beyond the window period and up to the steady state as follows:

For $$t_w \le t \le t_s$$:

$$\begin{aligned} VL(t)=VL(t_w)+\frac{C_w}{t}\exp \left( -\frac{(ln(t-t_w)-a)^2}{b}\right) \ , \end{aligned}$$

where $C_w$, *a*, and *b* are infection-specific calibration parameters. In this study, we assume that the viral load reaches steady state at time $t_s$, beyond which it remains constant at a level of $VL(t_s)$ (i.e., $VL(t)=VL(t_s)$, $\forall t> t_s$); this assumption can be easily relaxed. We note that the steady state viral load, denoted by $VL(t_s)$, can equal zero for acute infections, or can remain at some positive level for chronic infections. Consequently, the complete viral load model follows:

1 $$\begin{aligned} VL(t)= {\left\{ \begin{array}{ll} C_0 2^{t/\lambda }, &{} \text {if }t \le t_w\\ VL(t_w)+\frac{C_w}{t}\exp \left( -\frac{(ln(t-t_w)-a)^2}{b}\right) , &{} \text {if } t_w<t \le t_s\\ VL(t_s), &{} \text {if }t > t_s, \end{array}\right. }. \end{aligned}$$

where the window period component, $C_0 2^{t/\lambda }$, is the doubling time model in Busch et al. [6], with infection-specific calibration parameters $C_0$ and $\lambda$, where $\lambda$ represents the viral load doubling time within the window period. For demonstration, Fig. 1 plots the base 10 logarithm of the HIV viral RNA load, obtained by Eq. (1), versus post-exposure time in HIV-infected subjects, calibrated as discussed in "Viral load progression model" section.

#### Pooling dilution model

Pooled testing may reduce the test’s sensitivity due to pooling dilution, that is, the biomarker load of an infected specimen is diluted by infection-free specimens in the pool so that the infected specimen may no longer be detectable by the pooled test [22]. In this section, we model the test sensitivity considering pooling dilution. For this purpose, we first describe the probit function, proposed in the literature to model pooling dilution, and discuss how it is expanded to consider the number of infected specimens in a pool.

Towards this end, let $\tau \in \mathfrak {R}^+$ denote the life-time of a certain infection, and $n \in Z^+$ denote the testing pool size. We also let $T^+ (n)$ denote the event that the test outcome is positive for a pool of size *n*, indicating the presence of at least one infected specimen in the pool; and let $N_I(n)$ denote the number of infected specimens in the pool, which is a random variable with possible values $\{1, \ldots , n\}$. Therefore, the test sensitivity for pool size *n* (*Sens*(*n*)), that is, the probability that the test outcome is positive given that the pool contains at least one infected specimen, follows:

2 $$\begin{aligned} Sens(n) = P(T^+(n); N_I(n) \ge 1), \end{aligned}$$

where the “;” notation denotes probabilistic conditioning. Weusten et al. [27, 28] propose the following probit model to derive the sensitivity of pooled testing, under the assumptions that the test is perfectly reliable outside of the infection’s window period (i.e., $\tau = t_w$), and the pool contains at most one infected specimen, regardless of the pool size (i.e., $N_I(n) = 1$ with probability 1):

3 $$\begin{aligned} Sens(n) = \frac{1}{t_w} \int _0^{t_w} \Phi \left( z\dfrac{log\left( \frac{\chi C_02^{t/\lambda }}{n x_{50}}\right) }{log(x_{95}/x_{50})} \right) dt, \quad \hbox {(from [28])} \end{aligned}$$

where following [28], $\Phi (.)$ is the cumulative distribution function (CDF) of the standard normal distribution; *z* is a constant such that $\Phi (z) = 0.95$, i.e., $z=1.6449$; $\chi$ is the number of nucleic acid copies per viral particle, and $x_{50}$ and $x_{95}$ respectively denote the viral load measurement at which the probability of a pool testing positive is 50% and 95% [28].

We expand the probit model in Eq. (3) to consider the performance of a pooled test during the infection’s life-time, and to account for the possibility of multiple infected specimens in a testing pool. In particular, we first derive the test’s *conditional sensitivity* for a pool size of *n*, given that the pool contains *i* infected specimens, denoted by $Sens(n;i)=P(T^+(n); N_I(n)=i)$, $\forall i \in \{1,\ldots ,n\}, n \in {\mathbb {Z}}^+$:

4 $$\begin{aligned} Sens(n;i) = P(T^+(n); N_I(n) =i)=\frac{1}{\tau ^i}\underbrace{\int _0^{\tau }\int _0^{\tau }\cdots \int _0^{\tau }}_{i{\text {-fold}}} \Phi \left( z\dfrac{log\left( \frac{\chi \sum _{j=1}^i (VL(t_j)}{n x_{50}}\right) }{log(x_{95}/x_{50})} \right) dt_1dt_2\ldots dt_i, \end{aligned}$$

where $t_j$ denotes the (random) post-exposure time for infected specimen $j, j=1 \ldots ,i$, in the pool, and $VL(t_j)$ can be derived from Eq. (1). Observe that the probit model in Eq. (3) follows as a special case of Eq. (4), with $\tau =t_w$ and $N_I(n)=1$. Then, using the common binomial model for the number of infected specimens in a pool, and the law of total probability, the overall sensitivity of the pooled test, for pool size of *n* and infection prevalence rate of *p*, follows:

5 $$\begin{aligned} Sens(n) = \sum _{i = 1}^{n} Sens(n;i) P(N_I(n)=i) = \sum _{i = 1}^{n} Sens(n;i) \left( {\begin{array}{c}n\\ i\end{array}}\right) p^i (1-p)^{n-i}. \end{aligned}$$

On the other hand, the test’s *specificity* (true negative probability), given by:

$$\begin{aligned} Spec=1-P(T^+(n); N_I(n) =0),  \quad \text { } \forall n \in {\mathbb {Z}}^+, \end{aligned}$$

is independent of the pool size, because in the absence of infected specimens in the pool ($N_I (n) =0$), pooling dilution does not apply.

In summary, the proposed *sensitivity estimation model* in Eqs. (4)–(5) can be used in conjunction with Eq. (1) to determine the sensitivity of pooled testing for any pool size.

#### Calibration and validation

Calibration: We calibrate the sensitivity estimation model based on Stramer et al. [22], which provides the test sensitivity of an infected window period blood specimen diluted 16-fold (i.e., tested within a pool of size 16) as 88%. Therefore, $C_0$ is calibrated such that Eq. (3) equals 0.88 with $n=16$. Further, according to various studies, the HIV viral RNA load in blood peaks typically around day 17, with an average load of 6.8 $\log _{10}$ copies/ml, and reaches steady state around day 61, with an average load of 5.1 $\log _{10}$ copies/ml; the HIV doubling time ($\lambda$) is 0.85 days, and the number of nucleic acid copies per viral particle ($\chi$) for HIV is 2 [10, 20, 28]. Therefore, we calibrate the remaining parameters of our model, namely $C_w$, *a*, and *b*, in Eq. (1), based on these values; see Table 1 for the clinical data used and the calibrated parameter values. We note that this calibration is for demonstration purposes, and our model parameters can be calibrated for any given set of data.

**Table 1:** Calibration and validation data for the HIV and HIV ULTRIO Plus NAT Assay

Calibration data	
HIV viral RNA load data	
$t_w$	11 days [20]
$t_p$	17 days [20]
$t_s$	61 days [20]
$VL(t_p)$	6.8 $\log _{10}$ copies/ml [20]
$VL(t_s)$	5.1 $\log _{10}$ copies/ml [20]
$\lambda$	0.85 days [10]
$\chi$	2 copies/particle [28]
Test sensitivity data	
$P(T^+(16);N_I(16) =1, \tau = t_w)$	0.88 [22]
Calibrated model parameters	
$C_0$	9.000
$C_w$	$1.096 \times 10^8$
* a*	1.980
* b*	1.730
Validation data	
$Sens(n=1; N_I(1) = 1)$	(99.7–100%) [11]
$Sens(n=16; N_I(16) =1)$	(98.2–99.5%)) [11]

Validation: We validate our sensitivity estimation model using the overall (life-time) efficacy data for the HIV ULTRIO Plus NAT Assay, in terms of the 95% confidence interval (CI), published by the Food and Drug Administration (FDA); see Table 1. We use our model [Eqs. (1), (4), and (5)], with calibrated parameters reported in Table 1, to derive the conditional sensitivity values for the HIV ULTRIO Plus Assay for various pool sizes; see Table 2, which reports the derived conditional test sensitivity values as a function of the pool size and the number of infected specimens in a pool. According to Table 2, both $Sens(n=1;N_I(1) =1)= 99.98\%$ and $Sens(n=16;N_I(16) =1)= 99.26\%$ values are contained within the 95% confidence intervals reported by the FDA (see Table 1).

**Table 2:** Derived conditional sensitivity values for the HIV ULTRIO Plus Assay (in %) as a function of the pool size and the number of infected specimens in a pool (*Sens*(*n*; *i*)) (reported in 9 decimal point accuracy)

Pool size (n)									
Number of infected specimens (i)		1	2	3	4	5	6	7	8
1		99.98	99.93	99.88	99.82	99.76	99.70	99.65	99.59
2			99.9998	99.9995	99.9992	99.9988	99.9983	99.9979	99.9974
3				100.00000	99.99999	99.99999	99.99999	99.99998	99.99998
4					99.9999999	99.9999999	99.9999999	99.9999998	99.9999997
6						99.999999999	99.999999998	99.999999997	99.999999996
7							100.000000000	100.000000000	100.000000000
8								100.000000000	100.000000000

Pool size (n)									
Number of infected specimens (i)		9	10	11	12	13	14	15	16
1		99.54	99.50	99.45	99.41	99.37	99.33	99.29	99.26
2		99.9969	99.9963	99.9958	99.9953	99.9948	99.9942	99.9937	99.9932
3		99.99997	99.99996	99.99996	99.99995	99.99994	99.99994	99.99993	99.99992
4		99.9999997	99.9999996	99.9999995	99.9999994	99.9999993	99.9999991	99.9999990	99.9999989
5		99.999999995	99.999999994	99.999999992	99.999999991	99.999999989	99.999999987	99.999999985	99.999999983
6		100.000000000	100.000000000	100.000000000	100.000000000	100.000000000	100.000000000	100.000000000	100.000000000
7		100.000000000	100.000000000	100.000000000	100.000000000	100.000000000	100.000000000	100.000000000	100.000000000
8		100.000000000	100.000000000	100.000000000	100.000000000	100.000000000	100.000000000	100.000000000	100.000000000
9		100.000000000	100.000000000	100.000000000	100.000000000	100.000000000	100.000000000	100.000000000	100.000000000
10			100.000000000	100.000000000	100.000000000	100.000000000	100.000000000	100.000000000	100.000000000
11				100.000000000	100.000000000	100.000000000	100.000000000	100.000000000	100.000000000
12					100.000000000	100.000000000	100.000000000	100.000000000	100.000000000
13						100.000000000	100.000000000	100.000000000	100.000000000
14							100.000000000	100.000000000	100.000000000
15								100.000000000	100.000000000
16									100.000000000

As discussed above, the overall test sensitivity at any prevalence rate, *p*, can then be derived from the conditional sensitivity values in Table 2 via the law of total probability; see Eq. (5). As expected, conditional test sensitivity decreases with pool size, and increases with the number of infected specimens in a pool. Moreover, we observe that test sensitivity rapidly approaches 1 as $N_I(n)$, the number of infected specimens in a pool of size *n*, increases, and for $N_I(n) \ge 4$, test sensitivity becomes almost perfect.

We also derive $P(T^+(n);N_I(n) =0)=0.07\% =1-Spec$, i.e., $Spec = 99.93\%$, which is also consistent with the efficacy data for the HIV ULTRIO Plus NAT Assay, published by the FDA [11].

### An approximation for sensitivity estimation

Our model in "Pooled sensitivity estimation methodology" section derives the conditional test sensitivity values, *Sens*(*n*; *i*), and uses the law of total probability, along with higher dimensional integrals (up to pool size), to derive the overall (unconditional) test sensitivity values for a wide range of pool sizes. Thus, it can be computationally expensive, especially for large pool sizes. Therefore, in this section, we provide an approximation function for computing the pooled test sensitivity,which does not require higher dimensional integrals. We do this by fitting a function to the sensitivity data derived in Table 2 via linear regression so as to minimize the mean squared error (MSE) of the proposed approximation.

Consider the following functional form for conditional test sensitivity for pool size *n*, given *i* infected specimens in a pool:

6 $$\begin{aligned} {\widetilde{Sens}}(n;i) = 1-\beta \alpha ^{\left( \dfrac{i}{n^{\gamma }}\right) }, \; \ i \in \{0,1,\ldots ,n\}, n \in {\mathbb {Z}}^+, \end{aligned}$$

where $\alpha$, $\beta$, and $\gamma$ are calibration parameters. In particular, by definition of pooling dilution, the probability of detection reduces with pool size, implying that $\gamma \ge 0$ and $\alpha \in [0,1]$; and $P(T^+(n);N_I(n)=0)=1-Spec$ (see "Pooling dilution model" section), implying that $\beta =Spec$. The remaining parameters (i.e., $\alpha$ and $\gamma$) are derived so as to minimize the MSE between the fitted function and the data in Table 2, that is:

$$\begin{aligned} (\alpha ^*,\gamma ^*)={{\,\mathrm{arg\,min}\,}}_{\alpha ,\gamma }\left( \sum _{n=1}^{16}\sum _{i=0}^{n}\left[ Sens(n;i) - {\widetilde{Sens}}(n;i)\right] ^2\right) , \end{aligned}$$

**Fig. 2:**
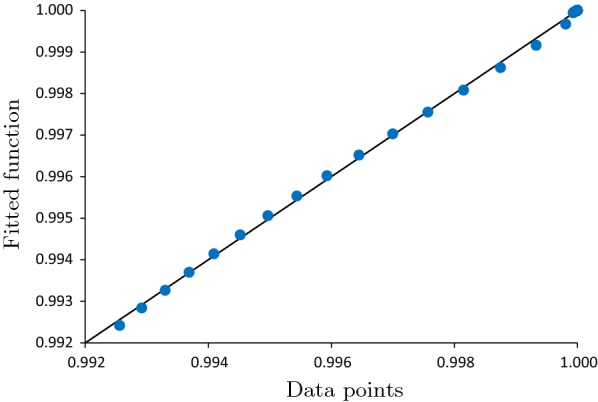
Fitted function versus the data points in Table 2

This minimization problem is a non-convex optimization problem, which we solve numerically in Python for the HIV ULTRIO Plus Assay, obtaining $(\alpha ^*=0.00033,\gamma ^*=0.179)$. The goodness of fit, measured by the coefficient of determination (i.e., $R^2$), is equal to 0.9995, suggesting that the fit is highly accurate; see Fig. 2 for the fitted model versus the data points in Table 2.

## Results

We apply our sensitivity estimation models (both exact and approximation models, respectively detailed in "Pooled sensitivity estimation methodology" and "An approximation for sensitivity estimation" sections) to determine an optimal testing pool design for HIV prevalence estimation in Sub-Saharan Africa. Specifically, we use the methodologies proposed in Pooled sensitivity estimation methodology" and "An approximation for sensitivity estimation" sections, along with the calibrated parameters in "Calibration and validation" section, to derive sensitivity estimates for the HIV ULTRIO Plus Assay for various pool sizes; and use these sensitivity values as inputs to a testing pool design optimization model, studied in the literature [17, 19, 24, 30].

### Testing pool design optimization

The optimization model determines an optimal testing pool design for prevalence estimation, in terms of the number of testing pools to be utilized, *s*, and the size of each testing pool, *n*, under a testing budget constraint, so as to minimize the *asymptotic variance* of the *maximum likelihood estimator* (MLE) of the unknown prevalence rate [19]:

7 $$\begin{aligned} \begin{aligned} \underset{n,s}{\text {minimize}}& \, \sigma ^2(n,s;p_0) \\ \text {subject to}& \, c_f \ s + c_v \ s n \le B \\& n \le \overline{N} \\&n, s \in {\mathbb {Z}}^+, \end{aligned} \end{aligned}$$

where $\sigma ^2(n,s;p_0)$ denotes the asymptotic variance of the MLE for a pool design (*n*, *s*), given an initial estimate of the unknown prevalence rate *p*, which we denote by $p_0$. The testing cost consists of a fixed testing cost per pool (e.g., cost of the testing kit), denoted by $c_f$, and a collection cost per specimen (e.g., cost of drawing blood), denoted by $c_v$. The tester has a total testing budget of *B* for prevalence estimation. Additionally, the maximum pool size that can be used may be restricted due, for example, to technological constraints, regulations, or other considerations, and we denote the maximum allowable pool size by $\overline{N}$. The asymptotic variance is a commonly used criterion for optimal testing design in prevalence estimation and for evaluation of estimators in statistical inference, and is also related to the Fisher’s information (e.g., [14, 17, 23–25, 30, 31]).

In pooled testing, only one test is used on each pool, and the test provides a binary outcome, with a positive outcome indicating the presence of at least one infected specimen in the pool; and a negative outcome indicating that all specimens in the pool are infection-free. Using the test outcomes, the tester derives the MLE of the unknown prevalence rate (${\hat{p}}$). In particular, for a given testing design, (*n*, *s*), let $S_I(s)$ denote the number of positive-testing pools among *s* pools, which is a random variable prior to testing. Then, after the testing is conducted and a realization of $S_I(s)=k$ is observed, the MLE of the prevalence rate corresponds to the value of *p* that maximizes the following likelihood function:

8 $$\begin{aligned} \begin{aligned} L (p; S_I (s)= k)&= \left( {\begin{array}{c}s\\ k\end{array}}\right) \Big [Sens(n;p) - (1-p)^n (Sens(n;p) + Spec - 1) \Big ]^{k} \Big [ 1 - Sens(n;p) + (1-p)^n (Sens(n;p) + Spec - 1) \Big ]^{s-k} \\&\Rightarrow {\hat{p}} \equiv \underset{p\in (0,1)}{\text {argmax}} \Big \{ L(p; S_I (s) = k) \Big \}. \end{aligned} \end{aligned}$$

The asymptotic variance function, $\sigma ^2(n,s;p)$, for a pool design of (*n*, *s*), and with respect to the unknown prevalence rate, *p*, is then given by (e.g., [17]):

9 $$\begin{aligned} \sigma ^2(n,s;p) = \frac{\left\{ Sens (n;p) - (1-p)^n (Sens (n;p) + Spec - 1) \right\} \left\{ 1 - Sens (n;p) + (1-p)^n (Sens (n;p) + Spec - 1) \right\} }{sn^2(1-p)^{2(n-1)} (Sens (n;p) + Spec - 1)^2}. \end{aligned}$$

### Study design and data

Our goal is in this section is to demonstrate the value of the sensitivity estimation methodologies developed in this paper through a numerical study. We do this by designing an optimal testing pool, based on the sensitivity estimates derived for the HIV ULTRIO Plus Assay for various pool sizes using the methodologies described in "Methods" section; and comparing the efficiency of the *optimal testing design* with a *benchmark design* that does not consider pooling dilution (hence does not need to use our methodology for sensitivity estimation at various pool sizes). As discussed above, we consider pool design for prevalence estimation of HIV in Sub-Saharan Africa using the HIV ULTRIO Plus Assay.

Model parameters are as follows. We assume that the actual prevalence rate is $$p = 0.044$$ [29], which is unknown to the tester; this prevalence rate is representative of the HIV prevalence rate in Sub-Saharan Africa. In the absence of this information, the tester determines an initial estimate of $$p_0 = 0.022$$, i.e., we consider the case of undershooting. Based on published data, we consider a fixed testing cost per pool of $31.5 [15], a collection cost per specimen of $8 [8], and a total testing budget of $5575 [18], which corresponds to a testing budget of 50 pools, each of size 10. Finally, we consider a maximum allowable pool size, of $$\overline{N}=48$$ [21]. These parameter values are for demonstration purposes, and one can conduct similar analyses with different parameter values.

As sensitivity inputs, we utilize the sensitivity values in Table 2, which are derived by the sensitivity estimation model in "Pooled sensitivity estimation methodology" section, in conjunction with the calibration parameters in "Calibration and validation" section. The sensitivity values in Table 2 correspond to pool sizes of $n=\{1, 2, \ldots , 16\}$. As discussed above, the sensitivity estimation model in "Pooled sensitivity estimation methodology" section requires the computation of higher dimensional integrals (up to pool size), and can be computationally expensive. Therefore, we use the approximation in "An approximation for sensitivity estimation" section to derive the sensitivity values for the remaining pool sizes, i.e., $n=\{17, \ldots , 48\}$. Then, we perform a two-dimensional search, over all possible values of $\{ (n,s): n \in \{1, \ldots , 48 \}, c_f \ s + c_v \ s n \le B \}$, to determine the optimal testing pool design, i.e., $(n^*, s^*)$, for the optimization model in Eq. (7) that minimizes the asymptotic variance. To determine the “best” benchmark design, we repeat the two-dimensional search, but without considering pooling dilution, that is, by replacing the parameters, *Sens*(*n*), $\forall n \in {\mathbb {Z}}^+$, with $99.98\%$, i.e., the sensitivity of individual testing for the HIV ULTRIO Plus Assay; see Table 3 for the resulting optimal design and the benchmark design. For each of these designs, we perform a Monte Carlo simulation to derive estimates for the MLE of *p*, ${\hat{p}}$ (see Eq.(8)); mean squared error (MSE); and the relative bias (rBias (%)), given by:

10 $$\begin{aligned} MSE = ({\hat{p}} - p)^2, \text { and } rBias(\%) = 100 \times \left| \frac{{\hat{p}} - p}{p}\right|. \end{aligned}$$

These performance metrics relate to the efficiency of prevalence estimation, and are commonly used in the literature, e.g., [13, 14, 30].

**Table 3:** Estimation efficiency (*mean* ± *half-width of 95% CI*) of the optimal design and the benchmark design for HIV prevalence estimation (with an actual prevalence rate of $p=0.044$)

Performance metric	Optimal design	Benchmark design
Pool design	$n^* = 37, s^* = 17$	$n^* = 17, s^* = 33$
${\hat{p}}$ (MLE)	0.05204 ± 0.00036	0.03041 ± 0.00029
MSE $(\times 10^4)$	3.95 ± 0.11	4.00 ± 0.08
rBias (%)	18.26 ± 0.52	30.88 ± 0.48

In particular, for each testing design, we perform 10,000 simulation replications. In each replication, we randomly generate the infection status of each of the $n^* \times s^*$ specimens, where each specimen carries an infection with probability *p*; and is infection-free otherwise; and for each infected specimen, we randomly generate a post-exposure time from a Uniform distribution with support $[0,\tau ]$, and compute the viral load using Eq. (1) and the parameters of "Calibration and validation" section. Then, we randomly assign the specimens into $s^*$ pools, each of size $n^*$, and generate the binary test outcomes based on the test sensitivity model given in Eq. (4). Finally, we compute the MLE, MSE, and rBias for each replication using Eqs. (8) and (10).

### Numerical study results

Table 3 reports the average estimation efficiency of the optimal design and the benchmark design, over 10,000 simulation replications. All performance metrics are reported in the form of *mean ± half-width of 95% confidence interval (CI)*.

As indicated by Table 3, the optimal design outperforms the benchmark design, and the differences are statistically significant. The benchmark design yields especially high bias in comparison to the optimal pool design, mainly due to the assumption of no pooling dilution, leading to biased estimates of the unknown prevalence rate.

## Discussion

Pooled testing is commonly used in public health settings, for both screening and surveillance of diseases and infections. An accurate and tractable method to compute the sensitivity of a pooled test is extremely important in designing the optimal pooled testing scheme for these efforts. As pooled NAT assays are widely used to screen for diseases, several approaches are proposed in the literature to compute the sensitivity of pooled NAT assays. However, these approaches only account for the window period of the infection, and assume perfect sensitivity past the window period, which is a restrictive assumption, especially as pooling dilution plays an important role in the sensitivity of pooled tests. Further, these studies compute the sensitivity of the pooled test based on the assumption of having at most one infected specimen in any testing pool, when the probability of having multiple infected specimens in a pool is, in fact, a function of both the pool size and the prevalence of the disease.

In this paper, we relax the restricting assumptions in the aforementioned studies and propose both exact and approximate models for computing the sensitivity of a pooled test. We expand the doubling time viral load model [6] to mathematically model the various growth phases of an infection; and propose an exact method to compute the conditional sensitivity of a pooled test as a function of the number of infected specimens in the pool and the pool size, by expanding the probit model in [27, 28]. Then, we can use a binomial model for the number of infected specimens in a pool, along with the law of total probability, to calculate the overall sensitivity of the pooled test given the pool size and the prevalence rate of the disease. We calibrate and validate our exact model using published data on the HIV ULTRIO Plus Assay. Finally, we propose an alternative approximation model to derive the sensitivity of pooled testing that is highly accurate and more analytically tractable than the exact method. We demonstrate the value of our exact and approximate models of pooled testing sensitivity in a case study on HIV prevalence estimation. In particular, we incorporate the proposed models into our testing pool design procedure for prevalence estimation of HIV in Sub-Saharan Africa. Our results show that the sensitivity model is very accurate for the HIV ULTRIO Plus Assay, enabling the design procedure to yield efficient testing pool designs that significantly minimize the estimation error, in comparison to a pool design procedure that utilizes less accurate sensitivity values (i.e., assuming no pooling dilution).

## Conclusions

In summary, we develop exact and approximate models for computing the sensitivity of a pooled test by expanding upon the commonly used probit model in [27, 28], and relaxing various restricting assumptions, as we previously discuss. Our methodologies are computationally tractable and highly accurate, and can significantly improve the efficiency of testing pool design for prevalence estimation, as demonstrated by our case study, and for public health screening. We further note that the proposed sensitivity estimation methodology is not infection-specific, and can be calibrated with clinical and published data for any infection or disease, e.g., hepatitis B and C viruses. In addition to its application in prevalence estimation, this methodology can be used in conjunction with other optimization models to make optimal decisions for classification efforts (e.g., [3]), and can also be used for setting a classification threshold, i.e., for classifying a subject as infected versus infection-free for the disease in question. Further, as the expanded viral load model considers the life-time of the infection, in regard to the biomarker load in infected subjects, it allows for more precise sensitivity estimation if information is available about the population of interest, e.g., repeat blood donors have lower overall HIV prevalence rates, and, due to their donation history, one can infer which stage of the infection the donor would be in, if infected. Therefore, integrating the sensitivity estimation methodology proposed in this paper with such optimization models would be worthwhile extensions of this research.

## Data Availability

The datasets used and analysed during the current study are available from the corresponding author on reasonable request.

## Appendix

See Tables 4 and 5.

**Table 4:** Summary of notation

Notation used in viral load and sensitivity models	
*VL*(*t*)	Viral load of an infected subject at time *t* post-exposure
$t_w$	The time at which the window period ends
$t_p$	The time at which the viral load peaks
$t_s$	The time at which the viral load reaches steady state
$\lambda$	Doubling time of the viral load during the window period
$\tau$	The life-time of the infection
$C_0, C_w, a,b$	Infection-specific calibration parameters
$T^+(n)$	The event that the test outcome is positive for pool size *n*, $n \in {\mathbb {Z}}^+$
$N_I(n)$	Number of infected specimens in a pool of size *n*, $n \in {\mathbb {Z}}^+$
*Spec*	Specificity of a test (constant for any pool size)
*Sens*(*n*)	Sensitivity of a pooled test, with pool size *n*, $n \in {\mathbb {Z}}^+$
*Sens*(*n*; *i*)	Conditional sensitivity of a pooled test, with pool size *n*, given that the pool
	Contains *i* infected specimens, $i \in \{0,1,\ldots , n\}$, $n \in {\mathbb {Z}}^+$
$\Phi (.)$	The cumulative distribution function (CDF) of the standard normal distribution
*z*	A constant such that $\Phi (z)=0.95$, i.e., $z=1.6449$
$\chi$	The number of nucleic acid copies per viral particle
$x_{50},x_{95}$	Viral load measurement at which the probability of testing positive is 50% and 95%, respectively
${\widetilde{Sens}}(n;i)$	Approximate conditional sensitivity of a pooled test, with pool size *n*, given that the pool contains *i* infected specimens, $i \in \{0,1,\ldots , n\}$, $n \in {\mathbb {Z}}^+$
$\beta$, $\alpha$, $\gamma$	Calibration parameters for the approximation model
*MSE*	Mean squared error
Notation used in the case study (prevalence estimation)	
*s*	Number of testing pools
*n*	Pool size
$p_0$	An initial estimate of *p*
$c_f$	Fixed testing cost per pool
$c_v$	Collection cost per specimen
*B*	Total testing budget
$\overline{N}$	The maximum pool size that can be used
${\hat{p}}$	The maximum likelihood estimator (MLE) of *p*
$\sigma ^2(n,s;p)$	The asymptotic variance of the MLE for a pool design (*n*, *s*),
	Given a prevalence rate of *p*
$S_I(s)$	Number of positive-testing pools among *s* pools
*rBias*	Relative bias of the MLE with respect to *p*

**Table 5:** Summary of abbreviations

HIV	Human immunodeficiency virus
NAT	Nucleic acid amplification testing
CDF	Cumulative distribution function
FDA	Food and Drug Administration
MSE	Mean squared error
MLE	Maximum likelihood estimator
CI	Confidence interval
